# Signal Transducer and Activator of Transcription Proteins at the Nexus of Immunodeficiency, Autoimmunity and Cancer

**DOI:** 10.3390/biomedicines12010045

**Published:** 2023-12-23

**Authors:** Clifford Liongue, Mohamed Luban Sobah, Alister C. Ward

**Affiliations:** 1 School of Medicine, Deakin University, Waurn Ponds, Geelong, VIC 3216, Australia; c.liongue@deakin.edu.au (C.L.); mlsobah@deakin.edu.au (M.L.S.); 2 Institute for Mental and Physical Health and Clinical Translation, Deakin University, Waurn Ponds, Geelong, VIC 3216, Australia

**Keywords:** cancer, cytokine, immunity, inflammation, STAT1, STAT2, STAT3, STAT5, STAT6

## Abstract

The signal transducer and activator of transcription (STAT) family of proteins has been demonstrated to perform pivotal roles downstream of a myriad of cytokines, particularly those that control immune cell production and function. This is highlighted by both gain-of-function (GOF) and loss-of-function (LOF) mutations being implicated in various diseases impacting cells of the immune system. These mutations are typically inherited, although somatic GOF mutations are commonly observed in certain immune cell malignancies. This review details the growing appreciation of STAT proteins as a key node linking immunodeficiency, autoimmunity and cancer.

## 1. Introduction

The signal transducer and activator of transcription (STAT) proteins were initially discovered in the context of interferon (IFN) signaling, being identified as the major transcription factor utilized to induce IFN-responsive genes to enable antiviral immunity [1]. Subsequently, other related proteins were identified, ultimately comprising a family of seven members: STAT1, STAT2, STAT3, STAT4, the closely related STAT5A and STAT5B, as well as STAT6. Each of the STAT proteins has subsequently been shown to play important but distinct roles downstream of specific cytokines that are involved in the development and function of immune cells, with several STATs having additional functions outside of the immune system [2]. Here, we provide an overview of the structure, function and both the canonical and non-canonical mechanisms of action of these proteins and also describe their normal physiological roles. The various classes of STAT mutations identified in human immune cell diseases are then detailed, with a discussion of their clinical implications.

## 2. Structure and Function of STAT Proteins

**Figure 1:**
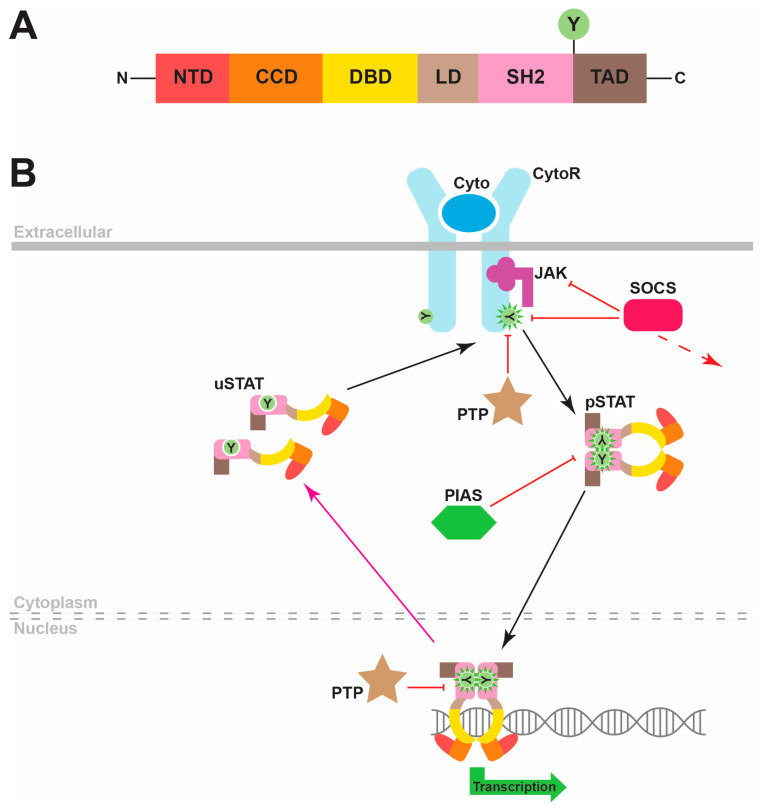
STAT protein structure and canonical function. (**A**). The common domain structure of STAT proteins, which comprises an N-terminal domain (NTD), coiled-coil domain (CCD), DNA-binding domain (DBD), linker domain (LD), SH2 domain (SH2) and transactivation domain (TAD), including a conserved tyrosine (Y) residue that is able to be phosphorylated. (**B**). Overview of the canonical mode of STAT signaling. Binding of a cytokine (Cyto, dark blue) to its specific cytokine receptor (CytoR, light blue) activates JAK kinases (purple), which is associated with the intracellular domains of these receptors that phosphorylate receptor tyrosine (Y) residues. STAT proteins are depicted in a schematic representation of their 3D structure with their respective domains being color-coded. These are initially latent, as cytoplasmic unphosphorylated STAT (uSTAT) molecules dock at these sites and become tyrosine-phosphorylated STAT (pSTAT) molecules that are mediated by the activated JAKs. These subsequently dissociate from the receptor and form pSTAT dimers that migrate to the nucleus to stimulate transcription of target genes encoding proteins impacting cell differentiation, proliferation, survival and function. Nuclear protein tyrosine phosphatase (PTP) proteins dephosphorylate pSTAT molecules to reform uSTAT molecules that are able to move back to the cytoplasm. Other negative regulators of the pathway include cytoplasmic PTPs that dephosphorylate cytokine receptor components, inducible suppressor of cytokine signaling (SOCS) proteins (green) that create a negative feedback loop by interfering with STAT docking, inhibiting JAKs and/or mediating degradation of cytokine receptor components, as well as protein inhibitors of activated STAT (PIAS) proteins that act via blocking STAT dimerization and nuclear entry.

STAT proteins possess a conserved six-domain structure that underpins the ability of these proteins to generate appropriate transcriptional changes in response to cytokines or other factors (Figure 1A) [3]. The N-terminal domain (NTD) facilitates the nuclear transport of these proteins, as well as contributing to the formation of STAT dimers through reciprocal interactions. The coiled-coil domain (CCD) mediates interactions with other proteins, which include negative regulators as well as other STAT proteins. The DNA-binding domain (DBD) enables the binding of STAT proteins to roughly palindromic DNA sequences that are present at specific sites adjacent to target genes across the genome to enable their transcriptional regulation. The DBD is connected to a unique linker domain (LD) that is presumed to provide structural support, which in turn connects to a Src homology 2 (SH2) domain. This protein interaction domain is common to a myriad of other signaling proteins and facilitates specific binding to phosphorylated tyrosine residues found on other proteins. In the case of STAT proteins, these are predominantly found in the intracellular region of cytokine receptors following cytokine binding. Additionally, the SH2 domain facilitates the formation of STAT homodimers and heterodimers via an interaction with a phosphotyrosine residue on a partner STAT protein. Finally, STAT proteins contain a highly variable transactivation domain (TAD) at the C-terminus, which mediates their ability to stimulate transcription through interactions with a range of transcriptional coactivators, as well as containing the critical tyrosine residue that is phosphorylated to enable STAT dimerization [3,4]. 

## 3. Mechanism of Action

The canonical mode of STAT signaling involves their major function as inducible transcriptional activators [4]. In this modality, unphosphorylated STAT (uSTAT) monomers remain in an inactive state in the cytoplasm until they are recruited to phosphotyrosine residues on cytokine and other receptors via their SH2 domain, which determines the cytokine specificity of the STAT activation (Figure 1B). Once docked, these uSTAT molecules in turn become tyrosine-phosphorylated, typically mediated by cytokine receptor-associated Janus kinase (JAK) proteins, thereby becoming “activated” tyrosine-phosphorylated STAT (pSTAT) molecules. These pSTATs then dissociate from the receptor and subsequently associate together to form dimers through reciprocal interactions of SH2 domains and phosphotyrosine residues on partner pSTAT proteins. Active pSTAT dimers then translocate into the nucleus, where they bind to specific DNA sequences to stimulate transcription of target genes. The consensus STAT recognition sequence is TTCN_2–4_GAA. However, the exact sequences recognized by each STAT protein differ from one another, providing a further layer of specificity [3,4]. 

The pSTAT proteins are inactivated by nuclear protein tyrosine phosphatases (PTPs), with the regenerated uSTATs returning to the cytoplasm [5,6]. The canonical STAT signaling pathway is also negatively regulated by cytoplasmic PTPs, which dephosphorylate various receptor components to inhibit STAT activation [7], by members of the suppressor of cytokine signaling (SOCS) family of negative feedback regulators, which are induced by STAT signaling and then act to inhibit further signaling via a number of mechanisms [8,9], and protein inhibitors of activated STAT (PIAS) proteins that can directly bind to STAT proteins to suppress nuclear entry. 

**Figure 2:**
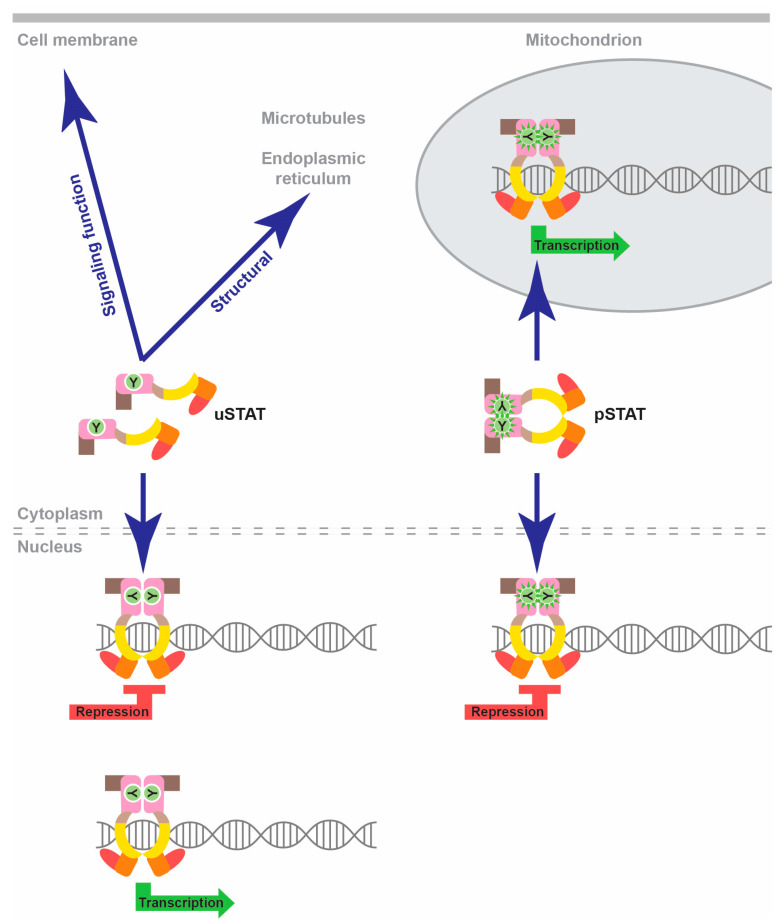
Non-canonical STAT functions. Schematic representation of non-canonical STAT functions, with pSTAT dimers able to facilitate nuclear transcriptional repression and mitochondrial transcriptional activation and uSTAT molecules with the potential to mediate both transcriptional activation and repression in the nucleus, as well as a variety of non-nuclear, non-transcriptional roles impacting the cell membrane, microtubules or endoplasmic reticulum.

However, it is important to note that alternative non-canonical modalities of STAT signaling exist [4,10] (Figure 2). Thus, pSTAT dimers can also facilitate transcriptional repression of specific nuclear genes, as well as mediate transcriptional activation of mitochondrial genes. Alternatively, uSTAT proteins can dimerize without the need for tyrosine phosphorylation, with some uSTATs also exhibiting DNA-binding capabilities [11]. In this manner, uSTAT proteins can facilitate both gene transcription and repression in the nucleus, but also perform additional non-nuclear, non-transcriptional functions impacting the cell membrane, microtubules and endoplasmic reticulum in particular cell settings [10,11]. Finally, alternate splicing and post-translational modifications such as serine phosphorylation, acetylation and ubiquitination can also impact the activity of specific STAT proteins in particular cellular contexts [4].

## 4. Physiological Roles of STAT Proteins

The majority of documented roles for STAT proteins in normal physiology relate to their canonical modality as inducible transcription factors that are responsible for switching on the expression of specific target genes. This is especially true in the immune system, where STATs serve as the preeminent mechanism for rapidly stimulating gene expression in response to external stimuli, principally cytokines that are active in immune cell regulation [2].

**Figure 3:**
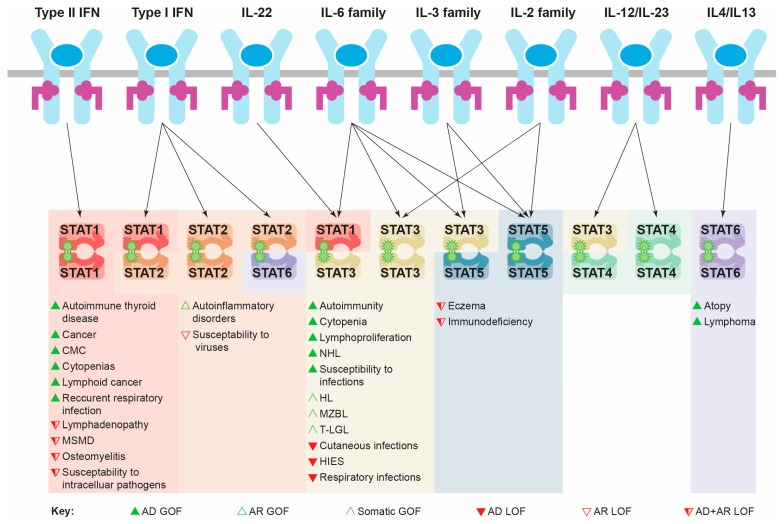
Specificity of STAT protein activation and impacts of different classes of mutation. Schematic of key immune cytokines activating specific STAT protein combinations in order to mediate appropriate transcriptional changes and cellular responses. The pathological consequences of the indicated mutational classes for each relevant STAT protein are shown below. Abbreviations: AD: autosomal dominant; AR: autosomal recessive; CMC: chronic mucocutaneous candidiasis; GOF: gain-of-function; HIES: hyper IgE syndrome; HL: Hodgkin lymphoma; IFN: interferon; IL: interleukin; LOF: loss-of-function; MSMD: Mendelian susceptibility to mycobacterial diseases; MZBL: marginal zone B cell lymphoma; NHL: non-Hodgkin lymphoma; STAT: signal transducer and activator of transcription; T-LGL: T cell large granular lymphocytic leukemia.

Individual STAT proteins have unique functions, playing critical roles downstream of specific cytokines (Figure 3). For instance, both STAT1 and STAT2 are principally involved in IFN signaling. The most significant roles of STAT1 relate to its ability to facilitate appropriate cellular changes in response to the entire family of IFN proteins [12], while the involvement of STAT2 is limited to responses downstream of IFNα and IFNβ, particularly mediating immunity to viral infections [12,13,14].

STAT3, by contrast, exerts its major functions downstream of the interleukin 6 (IL-6) family of cytokines, but is also activated by IL-22 and members of the IL-2 and IL-3 families. It plays a pivotal role in mediating leukemia inhibitory factor (LIF) signaling in stem cell self-renewal during early development [15]. STAT3 also plays a number of roles in T cell homeostasis, including facilitating IL-6-mediated survival [16] and impacting Th1/Th2 polarization and Th17 generation downstream of several cytokines [17,18,19]. It is also required for IL-2-mediated T cell proliferation [20,21], IL-10-mediated suppression of inflammation [17,22], as well as the mobilization, activation and emergency production of neutrophils, principally via a granulocyte colony-stimulating factor (G-CSF) [23,24].

STAT4 and STAT6 play more restricted roles, principally relating to the differentiation and polarization of T helper (Th) cells into either Th1 or Th2 subtypes by regulating interleukin (IL)-12/IL-23 and IL-4/IL-13 signaling, respectively [25,26], with additional functions in the control of neutrophil activation [27] and B cell fate [28].

Finally, the two STAT5 genes, STAT5A and STAT5B, show divergent functions despite a high sequence similarity. STAT5A is responsible for regulating lactation and differentiation of the mammary gland downstream of prolactin (PRL) signaling, whereas STAT5B is involved in growth hormone (GH) signaling impacting on growth and sexual dimorphism [29]. Both STAT5B and STAT5A play roles in immunity, including IL-2-mediated activation and differentiation of T cells, IL-7-mediated B cell development, as well as regulating the development and function of myeloid cells downstream of IL-3 family members like granulocyte macrophage-colony-stimulating factor (GM-CSF), as well as other cytokines [30,31]. 

## 5. Disruption of STAT Protein Function in Human Disease

A large number of human diseases impacting immune cells have been associated with the disruption of normal STAT protein functionality, particularly through mutations. These include both gain-of-function (GOF) and loss-of-function (LOF) germline mutations that yield a spectrum of inherited diseases from primary immunodeficiency to autoimmune diseases to immune cell cancers, as well as acquired GOF mutations observed in immune cell cancer and other malignancies (Table 1). These disorders are characterized by intriguing similarities as well as important differences between them, reflecting the distinct but overlapping nature of cytokine specificity (Figure 3), as well as the delicate balance that is required to maintain a healthy state [32,33]. In addition, single nucleotide polymorphisms (SNPs) in STAT genes have been associated with a similar range of diseases, while STAT hyperactivation mediated by mutations in other genes is also commonly observed in immune cell cancer and other cancers.

### 5.1. Germline Mutations

#### 5.1.1. STAT1 Mutations

Several distinct classes of germline STAT1 LOF mutations have been identified. These include complete LOF mutations with no functional protein and partial LOF with reduced levels of functional STAT1, both with autosomal recessive (AR) inheritance [46], although the partial LOF mutations can cause disease in compound heterozygous individuals [47]. In addition, LOF mutations with autosomal dominant (AD) inheritance have been described with typically normal STAT1 levels, but these proteins are dysfunctional and serve to abrogate wild-type STAT1 function [46,48], and so are perhaps better described as dominant–negative (DN) mutations. The complete LOF AR form results in defective signaling across all IFNs, whereas the partial LOF AR and LOF AD forms principally impact aspects of immunity that are mediated solely by IFNγ [46]. As a consequence, patients harboring all forms of STAT1 LOF mutations display an increased susceptibility to intracellular pathogens, including a Mendelian susceptibility to mycobacterial disease (MSMD) [34] and herpetic infection [32]. However, the disease presentation is milder in patients with the partial LOF AR form, where susceptibility to viral infections is normal, and those with the LOF AD form, which is essentially only associated with MSMD [46]. Other clinical phenotypes observed in STAT1 LOF patients are osteomyelitis and lymphadenopathy [34].

STAT1 GOF mutations are all AD and result in an increased responsiveness to both type I and type II IFNs, as well as IL-27 [46], as a consequence of more rapid and sustained STAT1 tyrosine phosphorylation and nuclear accumulation mediated by several mechanisms, including increased levels of STAT1 and other signaling proteins [46,49,50,51]. This results in augmented transcription of those genes that are typically activated, but also of additional genes [49,50,51]. This leads to patients presenting with disturbed IL-17 immunity, with significant Th17 cytopenia [36,48,50] with an associated impairment in IL-17A and IL-22 production [52], but also defects in B cell differentiation [53]. There is proinflammatory skewing, with reduced tolerogenic function of dendritic cells (DCs) [54], monocytes polarized toward a proinflammatory state with enhanced responsiveness to Toll-like receptor (TLR) 7/8 stimulation [55] and neutrophils with inflammatory markers [56]. Patients with these mutations represent around 50% of all cases of chronic mucocutaneous candidiasis (CMC), which is characterized by recurrent/persistent mucocutaneous infection by *Candida* fungi [36,57], but also exhibit increased susceptibility to bacterial, viral and other fungal infections of the lower respiratory tract, autoimmune manifestations, such as enterocolitis, systemic lupus erythematosus and relevant thyroid diseases [46]. These patients also have an enhanced cancer risk [36], including of Hodgkin lymphoma [58] and esophageal neoplasia [59], along with a propensity for cerebral aneurysm [32,46].

#### 5.1.2. STAT2 Mutations

Germline AR STAT2 LOF mutations, either homozygous or compound heterozygous, have been identified. This ablates the action of type I IFNs causing defective expression of interferon-stimulated genes (ISGs) and compromised antiviral induction, with STAT2 LOF patients exhibiting enhanced susceptibility to viral diseases, including potential susceptibility to attenuated viral strains used in vaccines [38,60]. These patients also display hyperinflammatory features, including hemophagocytic lymphohistiocytosis [38,60]. 

GOF STAT2 mutations are also AR, but in contrast results in hypersensitivity to type I IFN, which is mediated at least in part by preventing the USP18 protein from negatively regulating IFN receptor signaling, leading to prolonged phosphorylation of not only STAT2, but also STAT1 and JAK1 [39], with enhanced late IFN responses [61]. Patients that are homozygous for these mutations present with multisystem autoinflammation including neurological features that are typical of other type I interferonopathies [38,39]. 

#### 5.1.3. STAT3 Mutations

Germline STAT3 LOF mutations are also inherited in an AD manner, although many mutations are not familial and instead arise de novo [62]. These can affect the SH2 domain to interfere with dimerization or DBD to disrupt DNA binding, and can also act as DN mutants to reduce transcriptional responses mediated by wild-type STAT3 proteins [32]. These mutations result in impaired responses to IL-6, IL-10 and potentially IL-21 and IL-22 [32,63,64]. This results in defective Th17 cell production, reduced memory T and B cells, and impaired tolerogenic DCs and induced regulatory T (iTreg) cells [64,65], as well as elevated levels of circulating immunoglobulin E (IgE) antibodies that impact normal immune function [66]. Such STAT3 LOF mutations are a major cause of hyper IgE syndrome (HIES), particular the AD form, characterized by cutaneous and respiratory infections, mucocutaneous candidiasis and eczema, as well as skeletal muscle and connective tissue disorders [41,67]. These patients also exhibit an elevated risk of lymphoma, mainly non-Hodgkin lymphoma (NHL) [68].

STAT3 GOF mutations are also AD, and while their tyrosine phosphorylation is rarely enhanced, it is prolonged due to increased DNA binding and/or nuclear retention leading to increased transcriptional responses, some of which may negatively impact STAT5 activity [32,69]. These mutations cause decreased numbers and function of Treg cells in concert with perturbation of other T cell populations, including excess proliferation and defective CD8+ T cell tolerance [69,70,71], as well as disrupted differentiation of subsets of monocytes and myeloid DCs [72]. STAT3 GOF mutations are associated with early-onset multiorgan autoimmune disease, principally manifesting as arthritis and diabetes, as well as primary immunodeficiency that is associated with an increased susceptibility to recurrent severe infections [32,41,73]. In addition, patients also present with short stature, as well as increased risk of malignancy [32,41,74,75].

#### 5.1.4. STAT5B Mutations

A spectrum of germline STAT5B LOF mutations have been identified. There are classic AR forms, which are not phosphorylated and possess no transcriptional activity. Alternatively, there are AD DN forms, in which tyrosine phosphorylation occurs, but the mutants cannot enter the nucleus or fail to bind DNA, yet retain the ability to bind wild-type STAT5B and disrupt its normal transcriptional activity [76,77]. Collectively, these mutations are thought to abrogate aspects of signaling by IL-2, IL-15, growth hormone (GH) and potentially thymic stromal lymphopoietin (TSLP) [77,78,79,80]. This leads to a decreased number and functionality of Treg, gamma-delta T cells (γδT), CD8+ memory T cells and NK cells, along with B cell hyperactivity and elevated IgE [43,78,81,82] and reduced postnatal growth, with patients presenting with immunodeficiency and autoimmunity, characterized by chronic infections, diarrhea and eczema along with short stature, with patients with the AD form having milder symptoms, particularly with respect to immunodeficiency [77,80]. 

#### 5.1.5. STAT6 Mutations

STAT6 GOF mutations have recently been identified with AD inheritance, resulting in enhanced IL-4 responses, with sustained STAT6 phosphorylation and increased transcription of STAT6 target genes, augmented by a concomitant elevation in overall STAT6 protein levels [44,45]. Patients possessing these mutations display a strong Th2 skewing, which mediates early-onset atopic disease, including food allergy, eosinophilic asthma and atopic dermatitis [44], as well as an increased risk of follicular lymphoma [45]. 

### 5.2. Other Germline Variants

Single nucleotide polymorphisms (SNPs) in *STAT* gene loci have also been identified and are associated with a similar spectrum of immune cell diseases, including in *STAT4* and *STAT5A*, where disease-causing germline mutations have yet to be identified. Thus, *STAT3* SNPs have been associated with autoimmune thyroid diseases (AITD) [83], *STAT4* SNPs with early disease onset and severity of autoimmune diseases, including systemic lupus erythematosus [84] and rheumatoid arthritis [85], *STAT5A* SNPs with atopic dermatitis [86] and *STAT6* SNPs with asthma [87]. This extends to a susceptibility to immune cell cancers, with SNPs in *STAT3* and *STAT5A* being implicated in B cell lymphoma risk and SNPs in *STAT3* and *STAT6* in Hodgkin lymphoma risk [88].

### 5.3. Somatic STAT Mutations

Acquired somatic mutations in several STATs have also been identified and contribute to a variety of proliferative disorders and cancers [89]. However, it is important to note that these can be GOF mutations that are associated with oncogenesis or LOF mutations underpinning the ablation of a tumor suppressor role depending on the cellular context [89]. Somatic STAT3 GOF mutations have been identified as key driver mutations in T cell large granular lymphocytic leukemia (T-LGLL) and chronic NK lymphoproliferative disease (CLPD-NK), and STAT5B GOF mutations are also present in both T-LGLL and CLPD-NK at a lower frequency [42], as well as other T cell neoplasms [90] and myeloid neoplasm with eosinophilia and hypereosinophil syndrome (HES)/early onset eosinophilia [91,92], and has also been observed in chronic neutrophilic leukemia [93]. Somatic STAT6 GOF mutations are commonly observed in follicular lymphoma [94] and primary mediastinal large B-cell lymphoma [95].

### 5.4. Hyperactivated STAT Proteins

STAT proteins, particularly STAT1, STAT3, STAT5A and STAT5A, have been found to be hyperactivated in many cancers [96]. Indeed, STAT3 is considered an oncogene, being hyperactivated in approximately 50% of all human cancers [97,98], with its hyperactivation correlating with enhanced tumor progression [99] and poor prognosis [100,101,102]. Hyperactivation of STAT3 is particularly common in T-LGL [42] and of STAT5A in peripheral T cell lymphoma and leukemia [103], irrespective of their mutation status. STAT hyperactivation in such instances can be due to several factors, such as mutations in upstream regulators and high levels of inflammatory cytokines like IL-6 in the tumor microenvironment [99]. However, STATs can also be activated in other immune cell diseases. For example, STAT1 hyperactivation is associated with the immune dysfunction that is observed to be secondary to adenosine deaminase deficiency [104] and, indeed, in patients harboring STAT3 LOF mutations [105].

## 6. Clinical Implications of STAT Perturbations in Disease

The identification of causative STAT gene mutations has greatly impacted diagnosis of the related diseases. Thus, STAT1 GOF mutations have become diagnostic for AD-CMC [106], LOF mutations in STAT3 (and other components of the IL-6 signaling pathway) for HIES [64], and LOF mutations in STAT1 (and other components of the IFNγ signaling pathway) for MSMD [46]. However, given the symptom overlap between GOF and LOF mutations, functional analysis is necessary for precise diagnosis [46]—for example, analysis of IFN-induced STAT1 phosphorylation in patient PBMCs as an adjunct to genetic testing in interferonopathies [35].

**Figure 4:**
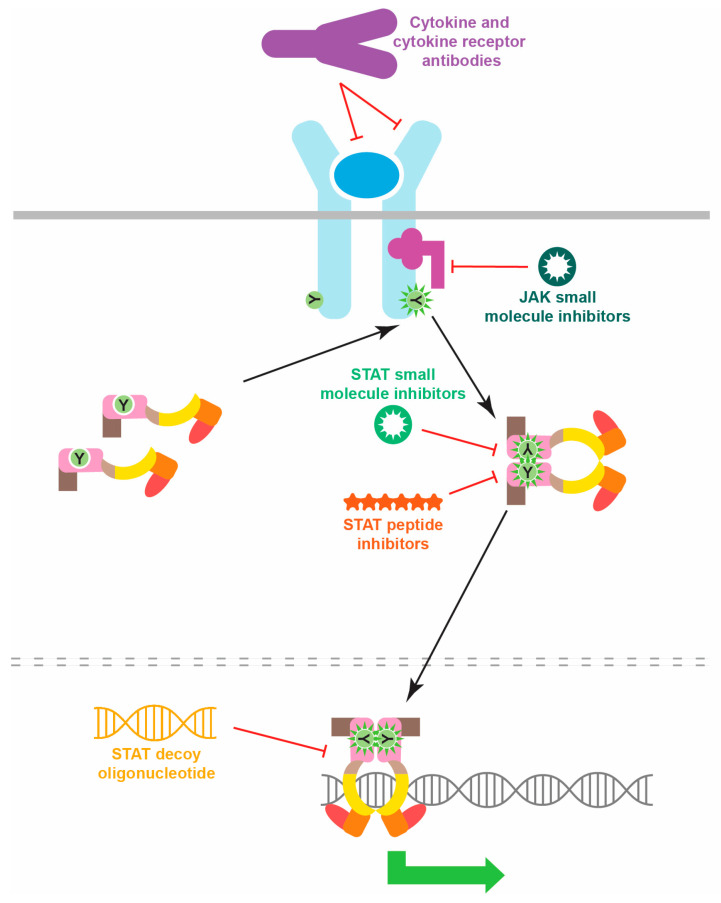
Strategies for inhibiting pathogenic STAT protein activation. Schematic representation of potential therapeutic classes able to inhibit inappropriate STAT activation, indicating their mechanisms of action.

The clear elucidation of specific STAT mutations has additionally underpinned a myriad of relevant possibilities for therapeutic targeting, especially for the GOF STAT mutations [107,108]. These therapeutics can be focused on multiple levels, from upstream cytokines, cytokine receptors and JAKs, to the STATs themselves, with agents targeting the latter being able to be directed toward particular functional aspects (Figure 4). For example, anti-IL-6R antibodies have shown promise in the treatment of STAT3 GOF disease [74] including malignancy [109], and anti-IL-4RA antibodies have demonstrated high effectiveness in the context of STAT6 GOF mutations [44]. Conversely, small-molecule JAK inhibitors have been demonstrated to reduce in vivo STAT activation [110] and improve clinical symptoms in patients harboring STAT1 GOF [34,57], STAT2 GOF [61], STAT3 GOF [111] or STAT6 GOF [45] mutations. Therapeutic agents have also been developed that directly target STATs, particularly small molecules and peptides directed to their SH2 domain to inhibit dimerization [112], oligonucleotide “decoys” to block DNA binding [113]. Alternatively, a number of strategies aim to reduce STAT protein levels, such as siRNA-mediated knockdown [114]. Several of the STAT inhibitors have shown effectiveness in vitro and in mouse tumor models [99]. A small molecule inhibitor that targets the phosphotyrosine-binding pocket of the STAT3 SH2 domain was able to block cell proliferation mediated by STAT3 GOF mutants [115]. However, the clinical reality has so far been that multiple therapeutics are typically required [41], or alternative strategies need to be employed, such as hematopoietic stem cell transplantation, including for patients harboring STAT1 GOF [57] or STAT3 GOF [69] mutations.

## 7. Conclusions

STATs are principally activated by the JAK kinases, named after the two-faced Roman god Janus, a fitting description from both a structural and functional perspective, where they also sit at the cusp of health and disease. It is perhaps then not surprising that STATs are also part of a similar Goldilocks paradigm, where either too little or too much STAT activity is able to alter the balance from a healthy state toward disease. This explains how both LOF and GOF variants can impact, including both germline and somatic mutations, particularly in the context of immune cells, where cytokine signaling via JAKs is especially important. However, it also provides a sound basis for the development of therapeutic approaches that can rebalance the equilibrium in a manner that might restore health in affected patients.

## Figures and Tables

**Table 1:** Mutations in STATs and their immune-cell-related clinical manifestation.

STAT Protein	Mutation Type	Immune-Cell-Related Manifestation	Key Signals Impacted	References
STAT1	LOF AR + AD	Susceptibility to intracellular pathogens and herpetic infection, MSMD, lymphadenopathy, osteomyelitis	↓ IFNs	[32,34]
	GOF AD	CMC, recurrent respiratory infection, cancer, autoimmune thyroid disease, cytopenias, lymphoid cancer	↑ IFNs, IL-27	[35,36,37]
STAT2	LOF AR	Susceptibility to viral disease	↓ type I IFNs	[38]
	GOF AR	Various autoinflammatory disorders	↑ type I IFNs	[38,39]
STAT3	LOF AD	HIES, cutaneous and respiratory infections	↓ IL-6, IL-10, IL-22	[32]
	GOF AD	Multiorgan autoimmunity, lymphoproliferation, cytopenias, susceptibility to infections and NHL	↑ IL-6 + others	[40,41]
	GOF somatic	T-LGL, HL, MZBL		[42]
STAT5B	LOF AR + AD	Immunodeficiency, eczema	↓ IL-2, GM-CSF	[32,43]
STAT6	GOF AD	Severe atopy, lymphoma	↑ IL-4	[44,45]

Abbreviations: AD: autosomal dominant; AR: autosomal recessive; CMC: chronic mucocutaneous candidiasis; GOF: gain-of-function; HIES: hyper IgE syndrome; HL: Hodgkin lymphoma; IFN: interferon; IL: interleukin; LOF: loss-of-function; MSMD: Mendelian susceptibility to mycobacterial diseases; MZBL: marginal zone B cell lymphoma; NHL: non-Hodgkin lymphoma; STAT: signal transducer and activator of transcription; T-LGL: T cell large granular lymphocytic leukemia; ↓: decreased; ↑: increased

## Data Availability

No new data were created or analyzed in this study. Data sharing is not applicable to this article.
